# Psychosocial Outcomes in StrokE: the POISE observational stroke study protocol

**DOI:** 10.1186/1471-2377-9-24

**Published:** 2009-06-12

**Authors:** Maree L Hackett, Nick Glozier, Stephen Jan, Richard Lindley

**Affiliations:** 1 Neurological and Mental Health Division, The George Institute for International Health, The University of Sydney, Sydney, Australia; 2 Psychological Medicine, The University of Sydney, Sydney, Australia; 3 Renal Division, The George Institute for International Health, The University of Sydney, Sydney, Australia; 4 Discipline of Medicine, Western Clinical School, The University of Sydney, Westmead Hospital, Westmead, Australia

## Abstract

**Background:**

Each year, approximately 12,000 Australians of working age survive a stroke. As a group, younger stroke survivors have less physical impairment and lower mortality after stroke compared with older survivors; however, the psychosocial and economic consequences are potentially substantial. Most of these younger stroke survivors have responsibility for generating an income or providing family care and indicate that their primary objective is to return to work. However, effective vocational rehabilitation strategies to increase the proportion of younger stroke survivors able to return to work, and information on the key target areas for those strategies, are currently lacking.

**Methods/Design:**

This multi-centre, three year cohort study will recruit a representative sample of younger (< 65 years) stroke survivors to determine the modifiable predictors of subsequent return to work. Participants will be recruited from the New South Wales Stroke Services (SSNSW) network, the only well established and cohesively operating and managed, network of acute stroke units in Australia. It is based within the Greater Metropolitan area of Sydney including Wollongong and Newcastle, and extends to rural areas including Wagga Wagga. The study registration number is ACTRN12608000459325.

**Discussion:**

The study is designed to identify targets for rehabilitation-, social- and medical-intervention strategies that promote and maintain healthy ageing in people with cardiovascular and mental health conditions, two of the seven Australian national health priority areas. This will rectify the paucity of information internationally around optimal clinical practice and social policy in this area.

## Background

The primary concern for many younger stroke survivors is returning to paid and unpaid work, not only for financial reasons, but to help rebuild confidence, regain independence, enhance recovery and reduce the social stigma of stroke.[1] However, the basis of, and targets for, effective vocational rehabilitation strategies are not understood. Data from the Auckland Regional Community Stroke (ARCOS) study revealed that only half of first time stroke survivors under the age of 65 years were in full time paid work at the time of their stroke.[2] This indicates that intervention strategies that improve return to all forms of work, including unpaid, are needed to obtain the greatest improvements in the personal wellbeing of stroke survivors.

A common misconception is that strokes affect only older people, however, one quarter (4.25 million people) of the estimated 17 million people worldwide who experienced a first-ever stroke in the year 2000 were under 65 years of age.[3] The number of Australians in this younger group alone is expected to have risen to 18,500 by 2017 as the decline in stroke incidence seen in older age groups has not been seen in those under 65 years of age in Perth and Auckland.[4,5] The small number of studies of younger stroke survivors have emphasised the social and economic toll of stroke including the high frequency of job loss, even in those with good functional recovery.[6] As younger stroke survivors live longer than older stroke survivors (82% of people under the age of 65 years in the ARCOS study were alive at one year after stroke compared with only 65% of those aged 65 years and over[7]) the ability to return to work will have long term personal benefits, in addition to reducing the burden on healthcare services, families and the global economy.

Stroke is already one of the most resource-intensive diseases affecting the population. The total cost of stroke in those aged less than 65 years in Australia in the year 2000 was $228 million. Around 10% (AU$23 million) of that overall cost was caused by reduced productivity because of stroke-related sick leave, early retirement and premature death.[8] Recent American estimates suggest that, with improved survival, loss of work and productivity are projected to be the major contributor to the lifetime cost of stroke.[9] The economic value of unpaid work e.g. paying for previously unpaid caring, is substantial,[10] and the positive effects of work (paid or otherwise) on subjective wellbeing is well recognised as providing an identity from which people derive meaning and satisfaction.

### What are the existing studies of return to work after stroke?

Before effective vocational rehabilitation strategies can be devised for younger stroke survivors, information is needed on barriers faced by this group of people, in particular, factors associated with return to work, factors which prevent people from returning to work, and factors which are modifiable. Unfortunately, the quality of existing studies is generally poor. A comprehensive review by Wozniak *et al *of the few available studies show a wide range in estimates of the proportion (from 9% to 91%) of stroke survivors who return to work[11] without identifying any targets for vocational rehabilitation strategies to improve the proportion able to return to work. Very few studies conducted time-to-return to work (survival) analyses to determine the factors that influence return to work and if they vary over time, nor did they distinguish been factors influencing return to paid work and unpaid work. The sample populations were highly selected, not generalisable, and methodological differences in length of follow-up, analytic strategies and definitions of work prevent the studies from being clinically informative.

One of the key issues pertaining to research on return to work is how the concept of 'work' is defined.[11] Some studies limited their analyses to those 'employed' before their stroke, others assessed resumption of normal activities (including school or household duties), some combined paid and unpaid work including study, while other studies excluded all forms of unpaid work. Few studies have determined the quality of return to work i.e. do survivors return to the same job for the same number of hours, the same job for fewer hours, do they return to a modified or new job or take up caring or home duties. The few studies published since the review do little to clarify the situation.

### The importance of psychosocial predictors of return to work

There is a paucity of information on potentially modifiable psychosocial predictors of return to work after stroke, especially in comparison with other cardiovascular diseases.[12] Studies of return to work following myocardial infarction have consistently found that psychosocial factors are important predictors.[11] In contrast, psychosocial factors have been considered in only a few stroke studies, where white collar workers[13] and those with higher education levels were found to be more likely to return to work in general,[11] and those with less demanding roles more likely to return after adjusting for stroke severity.[14] The most consistent predictors of return to work after stroke have been stroke severity (not modifiable) and dependency in activities of daily living (ADL).[11]

### Depression and work

Depression is generally characterised by some combination of abnormal thoughts, emotions, behaviour and relationships with others. It is associated with numerous poor outcomes including reduced quality of life,[15] increased use of healthcare resources, and greater functional disability than many chronic diseases.[16] Co-morbid depression also complicates the management of other cardiovascular disease by leading to higher rates of complications, longer length of hospital stay, and higher costs per episode.[17] In Organisation for Economic Co-operation and Development (OECD) countries poor mental health, primarily depression, is an increasing primary cause for leaving the workforce.[18] Recent research has highlighted the strong effect of poor mental health on leaving the workforce even amongst those with ostensibly physical conditions such as cardiovascular disease[19] and the additional work burden it places on those with co-morbid physical conditions.[20]

### Depression after stroke

Depression is commonplace after stroke, and often goes undetected and under treated. A systematic review and meta-analysis of the published observational studies of stroke incidence and outcome[21] indicates that on average one in three patients experience significant depression at some time after stroke, and that this risk is consistent over time. This review found that the proportion of depressed patients receiving any antidepressants after stroke in population-based studies ranged from 0% to 31%, which is similar to the 32% found to have received antidepressants in the ARCOS study,[22] indicating a potentially significant treatment gap. Depression has been shown to negatively impact on stroke outcome, including cross sectional associations with reduced health-related quality of life,[23] increased handicap[24] and subsequent mortality.[25] Extrapolating from other cardiovascular diseases it is likely that depression after stroke is negatively associated with returning to work. Currently, however, there is no evidence upon which to base a clinical trial, or to justify potentially costly changes to stroke rehabilitation and other services.

### The demands of work and economic hardship

Other psychosocial factors that have been implicated in determining return to work after cardiovascular disease include the relative demands of the job, support received at work, financial factors, private insurance, individual and family attributions, and education level.[26] These factors need to be systematically evaluated in stroke survivors. Recent studies across a number of countries have highlighted substantial societal costs follow stroke.[27,28] Households affected by an acute illness that results in ongoing health problems are likely to encounter previously unbudgeted expenses such as those associated with treatment, caring and day to day living e.g. the need for paid domestic help. It is not known whether such factors make stroke survivors determined to return to work before they are capable, or whether other household members to take on additional paid work. In either case, the psychosocial demands on the family would be increased and need to be quantified.

### Australasian data

Recent analysis of the ARCOS study was conducted to determine whether neurotic symptoms impact on return to paid work at six months after stroke. The ARCOS study is an 'ideal' stroke incidence study conducted in Auckland, New Zealand in 2002–2003, but it was not designed for the specific purpose of determining predictors of return to work. Only return to paid work was assessed and the only psychosocial assessment available at 28 days after stroke was the 28-item General Health Questionnaire,[29] a non-specific measure of a range of neurotic symptoms of clinical concern that produces a binary endpoint. Almost half of the younger stroke survivors who were in paid work before their stroke did not return to any paid work, with neurotic symptoms being an independent predictor of return to paid work (odds ratio 3.5; 95% confidence interval 1.7 to 7.3) after adjusting for stroke severity and other potential confounders.[2]

### Scientific rationale for a study of younger stroke survivors

While an increasing number of studies have been undertaken in the last decade focusing on stroke incidence, morbidity, mortality, acute therapies and primary and secondary prevention strategies, research on the outcome of stroke among those of working age has received little attention. The goal of rehabilitation for younger stroke survivors is to maximise the potential to return to independent living and gainful work and minimise economic hardship including the cost to society of ongoing treatment and lost productivity.

Stroke care and rehabilitation guidelines exist in several countries e.g. Australia, Canada, the United States of America and the United Kingdom. The Australian National Stroke Foundation guidelines for Rehabilitation and Recovery rely entirely on the review of the very limited studies[11] mentioned previously and recognise that "there is no evidence for interventions specifically to assist in returning to work." The United Kingdom National Service Framework and the Heart and Stroke Foundation of Ontario guidelines highlight the lack of evidence and practitioners in this area, while identifying vocational rehabilitation as one of the primary quality requirements. This demonstrates that any high quality research able to fill this evidence practice gap will be of international significance.

High quality research is needed to identify likely targets for intervention strategies aimed at improving the proportion of younger stroke survivors able to return to gainful work. Only a large adequately designed study focusing on return to work in younger survivors will resolve existing uncertainties. Such information is required to allow health services to be better configured to suit the needs of younger stroke survivors wishing to return to work, and to guide clinical practice. The Stroke Services New South Wales (SSNSW) network provides a unique platform to recruit a large and representative sample of younger stroke survivors with a broad spectrum of severity of stroke symptoms. The SSNSW is the only well established and cohesively operating and managed, network of acute stroke units in Australia. It is based within the Greater Metropolitan area of Sydney including Wollongong and Newcastle, and extends to rural areas including Wagga Wagga. These stroke units are linked through a Stroke Co-ordinating Committee. This resource is unique in Australia and represents a potential major clinical research platform.

## Methods/design

### Design and overview

POISE is a prospective multicentre observational study with study registration number ACTRN12608000459325. The primary aim of POISE is to determine if modifiable early (within 28 days of stroke) psychosocial factors are associated with people's ability to return to work one year after stroke. The secondary aim is to determine the economic impact of not returning to work for younger stroke survivors and their families. Participants are recruited as soon as possible after stroke from the Stroke Services New South Wales (SSNSW) network. Stroke status is verified by the recruiting centre staff and baseline demographic and clinical data are collected. Interviews are conducted by centrally located trained interviews at 28 days, 6 and 12 months.

### Study population

Recruitment began across the Stroke Services New South Wales (SSNSW) (see Table 2) network in Australia on 1 October 2008, and will continue until 440 participants are recruited.

Men or women aged over 17 years and less than 65 years of age will be eligible to participate in the study if they fulfil all of the following criteria:

1. Recent (within 28 days) acute stroke, defined according to the WHO standard diagnostic criteria of "rapidly developing clinical signs of focal (at times global) disturbance of cerebral function lasting more than 24 hours...with no apparent cause other than vascular origin",[30] this definition includes all main pathological subtypes, ischaemic stroke, primary intracerebral haemorrhage and subarachnoid haemorrhage (SAH) but excludes cases of silent stroke (detected by neuroimaging without appropriate clinical features) and transient ischaemic attack, and

2. Able to speak English sufficiently to respond to study questions, and

3. They or their proxy are able to provide written informed consent. (Participants with receptive aphasia or a severe language disorder or cognitive impairment (as determined by their treating clinician) are eligible to take part in the study provided their proxy is able to provide written informed consent and complete the assessments on the participant's behalf), and

4. Consent is given to contact the participant's general practitioner if necessary.

Study co-ordinators at each site review the medical records of potential participants to determine eligibility. Each recruiting site maintains a log of every eligible stroke patient who is offered participation in the study. The logs contain the initials and date of birth of the eligible patients and the date they were screened for participation in the study. Participants are asked to consent to the abstraction of information about their stroke from medical records, assessments at 28 days (baseline), six months and one year (primary endpoint), and for the participant's general practitioner (GP) to be contacted by research staff if necessary.

### Assessments and data collection

The detailed schedule for data collection is shown in Table 1.

**Table 1:** Schedule of assessments in the POISE study

**Participant Assessment ^a^**	**Screening**	**28 days^ b^**	**6 month**	**12 month**
Initials	X			

Date of birth	X			

Sex	X			

Inclusion criteria	X			

Contact information	X			

*Interview Characteristics*				

Method of assessment		X	X	X

Participant or proxy		X	X	X

Alive at scheduled time of assessment		X	X	X

Telephone Interview for Cognitive Status (TICS)[31]		X	X	X

*Demographic characteristics*				

Born in Australia		X		

Years lived in Australia		X		

Language other than English spoken at home		X		

Marital status		X		

Number of financially dependent children		X		

Social/financial living status		X	X	X

Height/Weight		X		

Highest educational qualification		X		

Pre-stroke social activities (Frenchay Activities Index)[32]		X		

*Pre-stroke Medical History*				

Smoking status		X		

Alcohol consumption: Audit Alcohol Use Disorders Identification Test (AUDIT-C)[33]		X		

Activity restricting illness		X		

Comorbidities		X		

Psychotropic medications		X		

Participated in talking therapy for mood		X		

*Current Medical History*				

Psychotropic medications			X	X

Participated in talking therapy for mood			X	X

*Pre-stroke Work Status*				

Lifetime occupation		X		

Most recent occupation		X		

Duration of most recent job		X		

Main income earner		X		

Union/organisation member		X		

Own own business		X		

Hours of paid, unpaid, domestic & volunteer work^c^		X		

Mode of travel to work		X		

Legally able to drive		X		

Drive for work		X		

Modified Job Content Questionnaire (JCQ)[35]		X		

Student		X		

*Current Work Status*				

Want to return to work		X	X	X

Returned to work: part time, full time		X	X	X

Returned to work: same conditions, altered conditions		X	X	X

Hours of paid, unpaid, domestic & volunteer work^c^		X	X	X

Legally able to drive		X	X	X

Mode of travel to work		X	X	X

Advised to stop driving since stroke		X	X	X

Driving since stroke		X	X	X

Student		X	X	X

*Pre-stroke household economics*				

Dependent on other household member for ADL		X		

Received carer payment		X		

Experiencing financial hardship		X		

Received financial assistance to meet costs		X		

Received government benefits		X		

Private health insurance		X		

Income protection insurance		X		

Household weekly income		X		

*Post-stroke household economics*				

Dependent on other household member for ADL		X	X	X

Received carer payment		X	X	X

Experiencing financial hardship			X	X

Received financial assistance to meet costs			X	X

Received government benefits			X	X

Private health insurance			X	X

Income protection insurance			X	X

Household weekly income			X	X

*Post-stroke social activities*				

Frenchay Activities Index[32]			X	X

*Post-stroke psychosocial Disability*				

WHO Disability Assessment Scale (WHODAS)[36]		X	X	X

*Post-stroke mood*				

Hospital Anxiety and Depression Scale (HADS)[37]		X	X	X

*Post-stroke sleep and vitality*				

Trouble sleeping, early waking, daytime tiredness		X	X	X

Snoring		X	X	X

SF-36 Vitality Scale[38]		X	X	X

*Rehabilitation*				

Physical, vocational, job assistance		X	X	X

*Social contacts*				

Close friends		X	X	X

Able to borrow money		X	X	X

Perception of social support		X	X	X

*Overall*				

Affect of stroke on life		X	X	X

^a ^Or proxy if applicable

^b ^Baseline assessment conducted at 28 days post-stroke

^c ^Questions modified from questions 34–51 of the Australian Bureau of Statistics 2006 census relating to jobs and work

Contact information, basic demographic characteristics and stroke information (subtype and severity) are collected by study co-ordinators at each site, immediately following consent, and entered into the internet-based case report forms (eCRFs).

At 28 days after stroke, consented participants are contacted, via telephone, by centrally located interviewers. This assessment includes collection of detailed demographic data, and pre-stroke medical history (prior vascular disease, pre-stroke disorders, other co-morbid conditions), social activities, work status and household economic status. At 28 days, six and 12 months after stroke information is also collected on current work, social and financial living status, use of interventions for mood problems, work status, driving status, household economics, social activities, psychosocial disability, mood, sleep, rehabilitation, social contacts and the overall affect of stroke. Each assessment begins with an assessment of cognitive status.

Specific questionnaires used include:

The *Telephone Interview for Cognitive Status *(TICS-M)[31] has been validated for assessment of cognitive function for research purposes. Scores are normally distributed and it is sensitive to change in cognitive performance. The 13-item TICS-M test includes orientation, recent and delayed memory, attention and comprehension assessments with a maximum score of 39. As the TICS-M is administered via the telephone it can be used in people with visual difficulties or poor hand-eye co-ordination.

The *Frenchay Activities Index *(FAI)[32] is a 15-item questionnaire specifically developed to measure social function in people with stroke. The FAI covers mainly domestic, leisure, social and work activities, and uses a four-point frequency scale ranging from 'never' to 'frequent' with total scores ranging from 0 (no activities) to 45 (full activities).

The *Alcohol Use Disorders Identification Test *(AUDIT-C)[33] uses three questions to collect information from the participants on 'at-risk' alcohol consumption. Individual question scores range from zero to four. At risk alcohol consumption is indicated by a total score of five or more for males or four or more for females. A single simple question is used to determine current *smoking *status.

Information on paid and unpaid work is collected using modified versions of questions 34–51 of the *Australian Bureau of Statistics 2006 Census *relating to jobs and work. [34] 2006 was the first year that questions on unpaid domestic work, caring for someone with a disability, looking after a child without pay and voluntary work were included in the Australian census. The same definitions and categories are used in POISE, where appropriate. The average number of hours of paid and unpaid work performed per week over the previous 12 months will be calculated as per the Australian census.

Participants are asked to indicate whether, and in what capacity, they have *returned to work *(paid and unpaid): same job, similar job, different job, average number of hours worked per week, not returned to work. The date of return to partial or full-time work is recorded. Questions are asked about changes in income and whether any government benefits (sickness, disability, or unemployment) or health-insurance benefits were received.

Specific barriers to return to work are determined using the short form of the *Job Content Questionnaire *(JCQ)[35]. This widely used measure assesses job demands, control over work and support received. JCQ scores have been shown to be associated with the risks of cardiovascular disease and return to work after cardiovascular disease. Participants are also asked to indicate if they are looking, or have recently looked, for work.

Psychosocial disability is assessed using the *World Health Organisation Disability Assessment Scale *(WHODAS II)[36]. This is a fully structured interview-administered assessment that is used to measure self-reported difficulty in functioning in six major domains: 'understanding and communicating' (six items), 'getting around' (five items), 'self-care' (four items), 'getting along with people' (five items), 'life activities' (eight items), and 'participation in society' (eight items). The WHODAS II standardised global score ranges from 0 (non-disabled) to 100 (maximum disability).

Information on depression and anxiety is collected with the *Hospital Anxiety and Depression Scale *(HADS);[37] a self report instrument, psychometrically validated in stroke survivors and specifically designed for use with medically ill patients. Scores of 8 (possible range 0–21) or more on the depression subscale are classified as 'depressed.' Consent has been obtained to convey scores of 8 or more directly to participants' nominated GP who will be permitted to arrange treatment or formal referral for depressive or other abnormal mood symptoms according to their clinical judgement. (The same procedure is followed for participants found to have depression or other abnormal mood symptoms of clinical concern at any other assessment point in the study.) Anxiety is similarly categorised.

Information on fatigue will be collected using the 'vitality domain (VT)' questions of the *Short Form 36 item *questionnaire (SF-36).[38]

*Economic hardship *is determined by a series of questions about failure to make household payments over the 6 months before stroke and whether there was help provided by any organisation or individual. An advantage of this measure is that it is sensitive to the possibility that individuals do prioritise certain payments (e.g. default on power bills to pay rent). The basis for these questions is the US Census Survey of Income and Program Participation.[39]

### Statistical considerations

To test the univariate association between depression at 28 days and return to work status at 12 months, a chi-squared test will be conducted. Assuming a conservative prevalence of depression of 25% and an overall return to work of 50% (as found in the ARCOS study[2]), then to detect a relative risk of returning work of 0.5 amongst the depressed we will require 150 participants. We will aim to recruit 220 participants to allow for loss to follow up, potential clustering effects, missing data and to provide sufficient numbers for multivariate modelling. We intend to examine return to paid and unpaid work separately so require a total sample of 440 participants.

### Statistical analysis

We will conduct a logistic regression model with 'returned to work' as a dichotomous response variable and a combination of *a priori *and univariately associated variables described in the 'assessments and data collection' as explanatory variables. The associations with the continuous outcome of the number of quality hours of work returned to will be modelled using linear regression.

The time of first return to work will be modelled by all the other explanatory variables using a Cox regression (survival analysis) model. These models will be fitted on two subsets of data: paid and unpaid work. The same predictor variables will be tested in each model and coefficients in the models for paid and unpaid work will be compared in relation to their size and significance to see how different variables affect return to paid and unpaid work, and time to return to paid and unpaid work.

Economic hardship will be constructed as a dichotomous variable where a reported inability to make any one of the payments posed to respondents will be classed as a case of economic hardship. A logistic regression model will be fitted to determine the extent to which variables affect the risk of this outcome as outlined above. Estimates of average production losses associated with cases of stroke, and stroke with depression as a complication, will be estimated based on an analysis of average amounts of time off work multiplied at the various rates for estimating production gains/losses outlined above. Adjustment for production losses caused by return to work at impaired capacity will be estimated. A sensitivity analysis will be conducted on this economic outcome involving the differing valuation methods and scenarios regarding the level of impairment and its impact on production.

### Ethical approval

Full ethical approval was received from the Human Research Ethics Committee (HREC) of the Sydney South West Area Health Service (SSWAHS) in May 2008: Protocol X08-0084, and from local institutional research governance offices for each clinical centre. Written informed consent is obtained for every participant. Proxy consent is permitted for patients unable to provide consent themselves to avoid exclusion of those patients with severe stroke.

## Discussion

This study addresses a key National Research Priority (ageing well, ageing productively) for a common disabling disease for young Australians. Support for those coping with the aftermath of stroke has been repeatedly stated as a major deficit of current stroke services, and a high priority for consumers. Research in the psychosocial aspects of stroke recovery fills a large gap identified by the National Stroke Foundation Rehabilitation and Recovery Guidelines[40] endorsed by the National Health and Medical Research Council of Australia in 2005.

It is anticipated that this study will, for the first time, reliably identify targets for early intervention and vocational rehabilitation strategies that will increase the proportion of younger stroke survivors who are able to return to a gainful work, here in Australia and internationally. Improving this outcome is considered of primary importance to younger stroke survivors, clinicians and policy makers and may have substantial economic benefits, quantified within this study, and direct implications for the long term psychosocial wellbeing of the many thousands of younger stroke survivors, and their families. An important feature of this study is the quantification of the social and economic consequences of stroke for the individual, society and the global economy, and identification of the key targets for intervention strategies to mitigate these effects.

This study has already generated academic and clinical interest, and support and involvement from health professionals and stroke survivors.

## Competing interests

The authors declare that they have no competing interests.

## Authors' contributions

MH is the principal investigator of POISE and is responsible for the day to day running of the study and drafted this manuscript. MH and NG jointly developed and wrote the protocol from its inception. SJ designed the economic analyses. RL is the chair of the Stroke Services New South Wales network research subcommittee which is the primary recruitment platform for POISE. MH, NG, SJ and RL are jointly responsible for the academic oversight of the study and all authors were involved in revising the manuscript and gave final approval for publication.

## POISE collaborative group

**Chief investigators**: Dr Maree Hackett, A/Prof Nicholas Glozier, A/Prof Stephen Jan, Prof Richard Lindley

**Associate investigators**: Prof Craig Anderson, Mr. Mark Longworth, Mrs. Judi Haliday, Dr Michael Pollack, Prof Sandy Middleton, A/Prof Louise Ada

**Project managers**: Ms Carol Burke, Mr Dan Jackson, Ms Marianne Byrne

**Research interviewers**: Ms Marianne Byrne, Ms Ruth O'Reilly

**PhD students**: Ms Beverley Essue

**Table 2:** POISE Collaborative Group

**Recruiting Centre**	**Name**	Role*
Armidale Hospital	Dr deGabriele	PI
Armidale Hospital	Ms Alex Little	SC
Bathurst Hospital	Ms Fiona Ryan	PI/SC
Blacktown Hospital	Ms Camelia Burdusel	SC
Blacktown Hospital	Dr Nigel Wolfe	PI
Campbelltown Hospital	Dr Chris Levy	PI
Campbelltown Hospital	Ms Chris Lyneham	SC
Concord Repatriation Hospital	Ms Alison Wilson	SC
Concord Repatriation Hospital	Associate Professor Alistair Corbett	PI
Dubbo Base Hospital	Ms Bonny Foye	PI/SC
Gosford Hospital	Mr Bryan Holder	SC
Gosford Hospital	Dr Jon Sturm	PI
John Hunter Hospital	Dr Michael Pollack	PI
John Hunter Hospital	Ms Sarah Moody	SC
Liverpool Hospital	Dr Darshan Ghia	PI
Liverpool Hospital	Dr Alan Mc Dougall	PI
Manly Hospital	Dr John Worthington	PI
Manly Hospital	Ms Tara Chambers	SC
Nepean Hospital	Mr Craig Harris	SC
Nepean Hospital	Dr Jonathan Wood	PI
Orange Hospital	Ms Fiona Ryan	PI/SC
Port Macquarie Base Hospital	Ms Kin Parrey	SC
Port Macquarie Base Hospital	Dr Matthew Kinchington	PI
Royal North Shore Hospital	Associate Professor Geoffrey Herkes	PI
Royal North Shore Hospital	Ms Susan Day	SC
Royal Prince Alfred Hospital	Ms Nadia Schweizer	SC
Royal Prince Alfred Hospital	Professor Craig Anderson	PI
St George Hospital	Dr Louise Allport	PI
St George Hospital	Ms Melissa Tinsley	SC
St Vincent's Hospital	Ms Naomi de Vries	SC
St Vincent's Hospital	Dr Romesh Markus	PI
Tamworth Rural Referral Hospital	Ms Rachel Peake	SC
Wagga Wagga Hospital	Dr Martin Jude	PI
Wagga Wagga Hospital	Ms Katherine Mohr	SC
Westmead Hospital	Dr Peter Landau	PI
Westmead Hospital	Ms Pip Galland	SC
Wollongong Hospital	Ms Michelle Doughty	SC
Wollongong Hospital	Dr Stephen Etheredge	PI
Wollongong Hospital	Dr Sundhar Rajan	SC
Wollongong Hospital	Dr James Roy	SC
Shoalhaven Hospital	Ms Susan Howard	SC/PI
Shoalhaven Hospital	Ms Kerrie O'Leary	SC
Wyong Hospital	Ms Justine Watkins	SC/PI

* PI: principal investigator; SC: site co-ordinator

## Pre-publication history

The pre-publication history for this paper can be accessed here:


